# Prostate Cancer Mortality in Iranian Men During 1990–2021: An Age-Period-Cohort and Joinpoint Regression Analysis

**DOI:** 10.1155/proc/8839773

**Published:** 2025-04-07

**Authors:** Fatemeh Jafari, Soheila Khodakarim, Fatemeh Baberi, Abbas Rezaianzadeh

**Affiliations:** ^1^Student Research Committee, Shiraz University of Medical Sciences, Shiraz, Iran; ^2^Department of Biostatistics, School of Medicine, Shiraz University of Medical Sciences, Shiraz, Iran; ^3^Colorectal Research Center, Shiraz University of Medical Sciences, Shiraz, Iran

**Keywords:** age-period-cohort effect, Iran, joinpoint regression, mortality, prostate cancer

## Abstract

**Background:** Prostate cancer (PC) ranks as the third cause of cancer-related deaths among Iranian men. The age-period-cohort (APC) model helps identify critical ages, periods, and high-risk birth cohorts to prevent and control PC. Thus, this research aimed to evaluate the effect of APC on PC mortality in Iran from 1990 to 2021.

**Method:** Our data include the number of PC deaths and population, collected by the Global Burden of Disease (GBD) and categorized by 5-year age groups. We computed average annual percentage changes (AAPCs) and relative risks by using joinpoint regression analysis and APC models, respectively.

**Results:** Crude and age-standardized mortality rates for PC were increasing, with AAPC of 2.254% (95% CI: 2.099% and 2.410%; *p* < 0.001) and 0.257% (95% CI: 0.088% and 0.428%; *p* < 0.001), respectively. Furthermore, an increase occurred in both age effect from ages 20–24 years (RR = 0.033; 95% CI: 0.023 and 0.046) to over 95 years (RR = 16.183; 95% CI: 14.702 and 17.814) and the period from 1992 (RR = 0.542; 95% CI: 0.516 and 0.570) to 2021 (RR = 1.892; 95% CI: 1.809 and 1.979). While, the cohort effect demonstrated a lower mortality rate in later born than earlier born (Coef = 2.302 for the < 1901 cohort compared to Coef = −2.249 for the 2002–2006 cohort).

**Conclusion:** Our study indicated that the trend of PC deaths in Iran increased during 1990–2021, and the period effect confirms this. Considering fewer deaths in high-income countries due to the widespread implementation of PSA testing, the occurrence of the aging phenomenon in our country, and the upward trend in deaths related to the age effect, sensitizing people and policymakers to conduct PSA screening seems necessary.

## 1. Introduction

Cancer is generally considered to be the leading cause of death worldwide. The global burden of cancer is rising rapidly, and known risk factors include smoking, poor diet, overweight, and lack of physical activity [1]. Prostate cancer (PC) is the second most common cancer worldwide and the fifth leading cause of cancer deaths in men, with approximately 1.5 million new cases and 397,000 deaths in 2022 [2]. The incidence of PC varies greatly between different world regions. The highest incidence was observed in Northern Europe (age-standardized incidence rate [ASR]: 83 per 100,000) and the lowest in South-Central Asia (ASR: 6.3 per 100,000). Regional mortality patterns do not follow the incidence pattern. The highest mortality rates are in the Caribbean, Sub-Saharan Africa, and Micronesia/Polynesia [3]. Changes in PC mortality patterns among countries may be related to the human development index (HDI), gross domestic product (GDP), life expectancy, ethnicity, prostate-specific antigen (PSA) screening, lifestyle, dietary habits, and environmental exposure [4–6]. According to the International Agency for Research on Cancer (IARC), PC is the most common cancer and the third leading cause of cancer deaths among Iranian men, with a mortality rate of 10.9 per 100,000 in 2022 [7].

Analyses of cancer incidence and mortality trends are often performed using age-adjusted rates, ignoring other effects [8]. Evidence has shown the overall probability of PC development increases with age, with a diagnosis of 1 in 22 men aged 60–69 years and 1 in 13 aged ≥ 70 years. PC is the third leading cause of cancer death in men aged 60–79 years and the second leading cause of cancer death in people aged 80 years or older [9]. Age-period-cohort (APC) models allow us to estimate age, period, and cohort effects and determine which one has the greatest impact on incidence and mortality [10]. Age effects reflect the biological and social processes of an individual's internal aging and developmental changes across the lifespan. Period effects are changes in time periods or calendar years that affect all age groups simultaneously. Cohort effects refer to changes in rates among people of a certain age but different years of birth [11]. Knowledge of current and future trends of PC in Iran is necessary to develop prevention, early detection, and treatment strategies that are important to improve patients' survival and quality of life. This process can guide decision-makers to prioritize resources in cancer services [12]. Therefore, the aim of this study is to estimate the effect of APC on PC mortality in Iran from 1990 to 2021.

## 2. Method

### 2.1. Data Sources

This study used the 2019 Global Burden of Disease (GBD) Survey dataset (https://ghdx.healthdata.org/gbd-results-tool), which includes parameters such as morbidity and mortality for 369 diseases and injuries in 204 countries and territories between 1990 and 2019 [13]. Complete details of the GBD methodology have been reported elsewhere [13]. Our study data include age- and year-specific PC deaths and population in Iran from 1990 to 2021.

### 2.2. Joinpoint Regression Analysis

First, we calculated the age-standardized mortality rate (ASMR) for PC using the standard population for low- and middle-income countries (INDEPTH) [14]. Then, to determine the magnitude of temporal trends in PC mortality, we used joinpoint regression analysis to assess the average annual percentage change (AAPC) and 95% CI. AAPC was calculated as the weighted geometric mean of different annual percentage change (APCh) values from the regression analysis [15]. The joinpoint regression model can be expressed as

(1) $$\begin{array}{c} E\left[y\left|x\right.\right]=\beta_{0}+\beta_{1}x+\sigma_{1}{\left(x-\tau_{1}\right)}^{+}+\cdots +\sigma_{k}\;{\left(x-\tau_{k}\right)}^{+}, \end{array}$$

where *y* represents the mortality of PC and *x* represents the year. The constant term in the model is denoted by *β*. *σ*, *τ*, and *k* represent the regression coefficients of each piecewise function, the unknown turning points, and the number of turning points, respectively. When (*x* − *τ*_1_) > 0, (*x* − *τ*_*k*_)^+^ = (*x* − *τ*_*k*_); otherwise, (*x* − *τ*_1_) = 0.

Each *p* value is found via Monte Carlo methods, and the level of overall asymptotic significance level is maintained through Bonferroni modification [15]. The analysis was conducted using “Joinpoint” software Version 5.0.2 from the Surveillance Research Program of the United States' National Cancer Institute.

### 2.3. APC Analysis

The APC model is a statistical analysis method commonly used in demographics, sociology, and epidemiology that follows the Poisson distribution. In our study, the APC model includes arranging data for mortality into 16 age groups, namely, 20–24, 25–29,…, 95 years, and older. To prevent overlapping of information between successive cohorts, the intervals for each period were set at 5 years, ranging from 1992–1996 to 2017–2021. As a result, 21 cohorts were created based on the 16 age categories and 6 period groups, which are categorized as < 1901, 1902–1906, and continuing through 1997–2001.

The APC can be generally expressed as follows:

(2) $$\begin{array}{c} Y_{ij}=\log\left(M\right)=\mu +{\alpha \text{ age}}_{i}+{\beta \text{ period}}_{j}+{\gamma \text{ cohort}}_{\text{⁣}k}+\varepsilon_{ijk}, \end{array}$$

where *Y*_*ij*_ is the PC mortality rate for the *i* th age group at the *j* th period, *μ* is the intercept of the regression equation representing the baseline level of risk of onset of disease or death, *ε*_*ijk*_ represents the term error, and *α*, *β*, and *γ* are the effects of age group, period group, and cohort group *i*, *j*, and *k*, respectively, where *i*, *j*, *k* = 1, 2,…

The main problem in the APC model is the “nonidentification problem,” which arises from an inherent mathematical relationship within the model. To overcome the nonidentification problem, we used the intrinsic estimator (IE) method because of its favorable statistical characteristics, such as providing unbiased estimates for small time periods and rate tables and not requiring the determination of a reference category for age, period, or cohort coefficients [16]. The coefficients were expressed in exponential form (exp(coef.) = *e*^coef.^), reflecting the relative risk (RR) of incidence and mortality associated with a specific age, period, or birth cohort in relation to an average level. For the analysis, the “apc_ie” command within STATA software Version 17.0 was used. Akaike information criterion (AIC), Bayesian information criterion (BIC), and variance were used to evaluate the degree of data-fitting diagnosis. The significance level was set at 0.05.

## 3. Results

**Figure 1:**
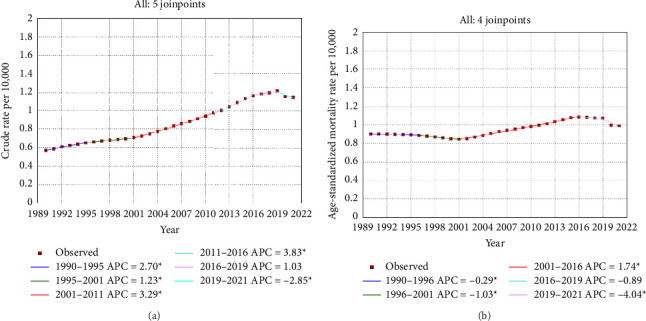
The Joinpoint analysis of crude (a) and age-standardized (b) mortality rates of prostate cancer among Iranian men during 1990–2021.

Joinpoint regression analysis revealed a crude mortality trend for PC across five intervals: 1990–1995, 1995–2001, 2001–2011, 2011–2016, 2016–2019, and 2019–2021, with a consistent increase noted in all periods except for the most recent one. To explain the APCh, the mortality rate during the 2011–2016 interval rose by 3.834% (95% CI: 3.452% and 4.218%; *p* < 0.001) each year, whereas the AAPC indicated that over the entire study duration, the mortality rate increased by 2.254% (95% CI: 2.099% and 2.410%; *p* < 0.001) annually (Table 1; Figure 1(a)). Moreover, ASMR declined at the four points where a breakpoint was noted, with the exception of the period from 2001 to 2016. However, the overall analysis indicated that ASMR rises by 0.257% annually (95% CI: 0.088% and 0.428%; *p* < 0.001) as shown in Table 1 and Figure 1(b).

**Figure 2:**
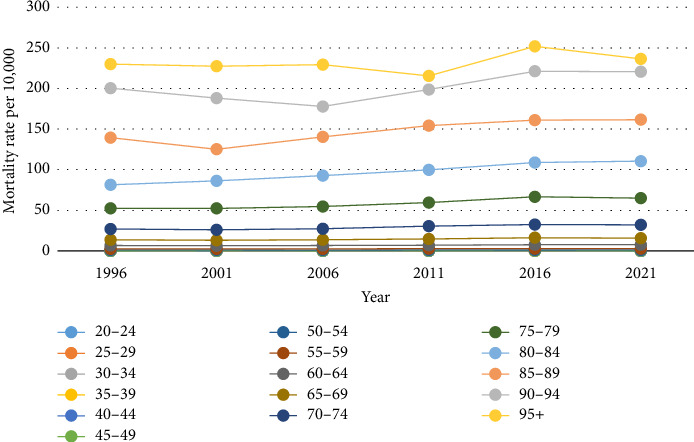
Mortality rate of prostate cancer among different age groups of Iranian men from 1990 to 2021.

Figure 2 indicates that across all age groups, there was a slight upward trend in PC mortality from 1992 to 2021, with the exception of 2001, 2006, and 2011 when a decline was noted among people aged 85–89, 90–95, and over 95, respectively. To understand this drop, if we subtract the age from the cohort year (for instance, 2001–89 = 1912, 2006–90 = 1916, and 2011–95 = 1916), we discover that these three declines are associated with those born in the cohort of 1912–1916, who saw a reduction in numbers for three straight years.

**Figure 3:**
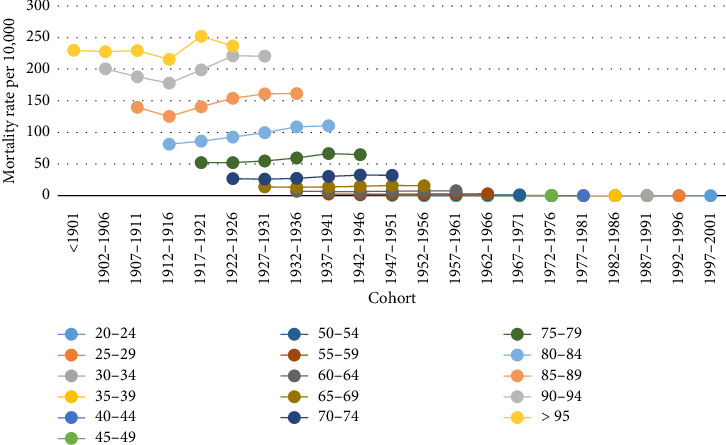
Cohort trends of the mortality rate of prostate cancer among the different age groups of Iranian men.

Cohort trends in PC mortality across various age groups are illustrated in Figure 3. It is evident that the mortality rate for younger cohorts is higher compared to older cohorts in each age group. Similar to what was presented in Figure 2, the cohort from 1912 to 1916 experienced a decline in the population aged 85 and older. Furthermore, there has been a reduction in the mortality rate among individuals over 95 years old during the most recent time period which is related to 2017–2021.

**Figure 4:**
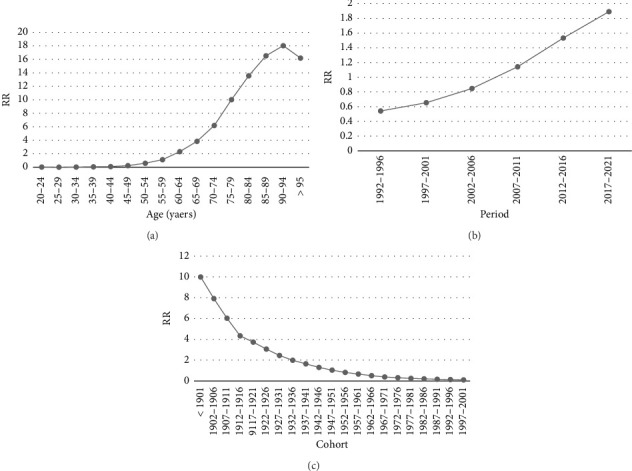
Prostate cancer mortality of Iranian men relative risks due to (a) age, (b) period, and (c) cohort effects.

As previously mentioned, we employed the APC model utilizing the IE method to calculate the coefficients related to age, period, and cohort effects. The findings from the APC model indicate that the age effect has risen, with the exception of the age group exceeding 95 years (Figure 4(a)). For instance, the coefficient reflecting the age effect increased from −3.409 in the 20–24 age category to 2.784 in the 95+ age category (Table 2). The trend of period effects has been on the rise, indicating that when adjusting for effects of age and cohort, the PC mortality rate increased from 1992 (RR = 0.542; 95% CI: 0.516 and 0.570) to 2021 (RR = 1.892; 95% CI: 1.809 and 4.198) (Table 2; Figure 4(b)), while the cohort effect has been decreasing (Figure 4(c)). This means that after adjusting for age and period effects, individuals born in more recent years exhibit significantly lower mortality rates compared to those born in earlier years. For example, the mortality rate for individuals born before 1901 is 94.72 times higher than that of those born between 1997 and 2001 (*e*^coef (〈1901)−coef (1997 − 2001)^) (Table 2) period. This interpretation can be utilized to compare each cohort group, age group, or period group. Moreover, it is important to highlight that the slope of the graph has decreased for those born in 1947–1951 and subsequent cohorts (Figure 4(c)). All of these analyses were significant at the 0.05 level.

## 4. Discussion

The current research utilized longitudinal data from the GBD to investigate how age, period, and cohort influence PC mortality and its trends in Iran from 1990 to 2021. Our joinpoint analysis indicated that both crude and ASMR for PC in Iran rose from 1990 to 2021 period. However, Wang et al. identified a downward trend in ASMR in China from 1990 to 2019 [17]. Previous research has shown that although high-income nations have experienced a decrease in mortality rates, low- and middle-income countries still face higher rates of PC–related deaths due to insufficient access to early diagnostic and treatment services [6]. In the European Randomized Study of Screening for PC (URSPC), a 20%–31% decrease in PC mortality with PSA screening was observed [18]. Furthermore, studies have indicated that HDI has a negative correlation with ASMR of PC [19]. It is to be noted that PSA screening has evolved significantly over recent decades. The United States Preventive Services Task Force (USPSTF) revised its guidelines in 2017, changing from a Grade *D* to a Grade C recommendation for men aged 55–69, emphasizing individualized decision-making based on potential benefits and harms. This change led to increased PSA testing rates, reversing earlier declines [20]. However, widespread PSA screening also poses challenges, including overdiagnosis and unnecessary treatments. The PSA test can detect cancers that grow so slowly that they would never cause symptoms, but treating them can lead to unnecessary complications such as urinary dysfunction and sexual issues [21]. Despite these challenges, well-articulated screening policies can help mitigate these risks while ensuring timely detection and management of high-risk cancers. In the context of rising PC mortality in Iran, adopting targeted screening strategies could be crucial in balancing early detection benefits with the need to avoid unnecessary interventions [22].Thus, due to Iran's growing trend in PC mortality, the observed increase in age and period affect mortality in the present study and the phenomenon of population aging, it is essential to adopt strategies to slow down or reverse this trend, such as pinpointing high-risk populations and promoting PSA screening through public health initiatives to balance early detection benefits with the need to avoid unnecessary interventions. However, there was a decrease in mortality between 2019 and 2021. One possible explanation is the insufficient identification and recording of cancer patients, leading to a reduction in documented deaths from this disease. In addition, in Italy, PSA testing saw a drop during the lockdown period [23]. Another reason for the decline could be the recording of deaths due to COVID-19.

In contrast to our research, which showed that all age groups faced a rise in PC mortality, a study conducted in China indicated that all groups experienced a decrease in mortality rates until 2019, with the exception of individuals aged 80 and above [24]. In addition, Figure 2 illustrates that among those over 95 years old, the mortality rate declined during the latter part of the observed period, which is linked to the growing elderly population and aging during 2017–2021 [25]. Our data support this hypothesis, as the population aged over 95, which serves as the denominator for the mortality rate, has risen compared to earlier periods. Consequently, the deductible value has decreased. Besides, a decrease was observed in the 85–89 age group in 2001, in the 90–95 age group in 2006, and in those over 95 in 2011. By subtracting age from the respective year (for instance, 2001–89 = 1912, 2006–90 = 1916, and 2011–95 = 1916), we can determine that these individuals were born between 1912 and 1916. There is a lack of adequate documentation for this decline, and further research is necessary concerning this birth group.

The APC model aids in identifying critical ages, time periods, and high-risk birth cohorts essential for the prevention and management of PC. In our research, the age effect indicated that mortality rates rose significantly with age, particularly among the elderly population. He et al. obtained results consistent with our findings [26]. Prior research has indicated that older men with high-risk PC tend to be at an advanced age at the time of their diagnosis and are frequently treated solely with androgen deprivation therapy [27, 28]. Furthermore, early-onset PC may present differently from cases diagnosed later in life with respect to both causes and prognosis [29]. In other words, older individuals generally have a poorer prognosis and higher failure rates compared to younger patients [30], because younger patients tend to have fewer comorbid conditions, while older patients typically face more [29].

The period effect is commonly influenced by a complex set of historical events and environmental influences, including wars, economic downturns, outbreaks of infectious diseases, public health interventions, and socioeconomic development [31]. The period effect demonstrates that there has been a rise in PC mortality rates over time. Ribeiro et al. obtained similar findings in the United States [32]. With changes in lifestyle, various elements can influence PC mortality rates, including elevated BMI, smoking [33], and alcohol consumption. Conditions such as diabetes, cardiovascular disease, and severe malnutrition also contribute significantly to PC mortality. Additional factors involve delayed diagnoses that complicate treatment for PC, which often progresses to a more advanced stage. The main causes of late diagnosis are insufficient healthcare resources, low health awareness, and flawed policies [34].

Analysis of cohort effect with the APC model indicated that younger cohorts had a lower mortality rate compared to older cohorts. However, in Figure 3, where cohort effect was not distinguished from age and period effects, findings were contrary, which could be attributed to the confounding influence of one or both age and period effects. In addition, the research conducted by Liu et al. supports the findings of our study in this regard [31]. A likely explanation for this is increased awareness of health and disease prevention, improved access to education [35], better treatment options, and a more active surveillance system in younger cohorts [36].

This study's strength is that it offers the first extensive national estimate of PC mortality in Iran over a 32-year period. In addition, the novelty of this research is found in the use of advanced trend models to analyze cancer mortality. Nonetheless, our study encountered some limitations. First, the GBD estimates were reconstructed using a wide variety of sources that varied in quality. Therefore, they need to be validated through epidemiological surveys conducted nationwide. Second, there was no available information regarding clinical staging, screening, and other specific characteristics of PC, which prevented us from assessing the impact of these factors in our analysis. Third, our findings cannot be generalized to every individual, as this would result in an ecological fallacy.

## 5. Conclusion

We conducted a comprehensive analysis of PC mortality trends among Iranian men from 1990 to 2021, utilizing APC modeling with IE and joinpoint regression. Our research indicated that the trend of PC deaths in Iran is increasing, and APC modeling with IE and joinpoint regression supports this finding. This approach provides significant insights into the interplay of age, period, and cohort effects on mortality rates, underscoring the robustness and reliability of these statistical tools. By effectively addressing the identification problem inherent in APC analysis, our study demonstrates the utility of these methodologies in uncovering complex temporal trends. The model's goodness-of-fit, validated through AIC and BIC diagnostics, further supports the soundness of our analytical framework. Beyond the context of PC in Iran, the application of these methodologies holds significant potential for investigating mortality trends and disease patterns across diverse populations and health outcomes. For instance, these tools can be applied to study chronic diseases such as cardiovascular diseases and diabetes, where understanding age, period, and cohort effects is crucial for developing targeted interventions. In addition, they can inform analyses of infectious diseases such as tuberculosis, where temporal trends are influenced by public health policies and environmental factors. The versatility of these methods also extends to mental health research, such as analyzing trends in self-harm rates, where cohort effects can reveal generational influences on behavior. Overall, this approach offers a diverse set of tools for public health research, enabling policymakers to adjust interventions based on subtle insights into demographic and temporal trends.

### 5.1. Suggested Future Research

Future studies should focus on the validity of GBD estimates through nationwide epidemiological surveys to ensure data accuracy. In addition, it is very important to gather detailed information about clinical stages and screening throughout Iran to understand their impact on patient outcomes. Examining the effectiveness of PSA testing in Iran and comparing its results in areas with and without extensive screening will also be beneficial to assess the impact of screening on reducing mortality. More research is needed on regional changes in PC incidence and mortality, as well as the role of lifestyle, genetic, and environmental factors. In addition, studying how population aging affects PC trends can inform healthcare adaptations. To improve documentation and data collection, standard protocols for PC data collection must be developed and health information systems must be integrated to facilitate data sharing. Evaluating public awareness campaigns and screening programs can also help increase early detection rates and reduce deaths. By addressing these areas, future research could provide more detailed insights into PC trends in Iran and inform effective public health strategies.

## Figures and Tables

**Table 1:** Crude and age-standardized mortality trends of prostate cancer among Iranian men based on joinpoint regression during 1990–2021.

Variable	Years	APCh (95% CI)	*p* value	AAPC (95% CI)	*p* value
Crude rate	1990–1995	2.702 (2.435, 2.970)	< 0.001	—	—
	1995–2001	1.232 (0.968, 1.496)	< 0.001	—	—
	2001–2011	3.286 (3.176, 3.396)	< 0.001	—	—
	2011–2016	3.834 (3.452, 4.218)	< 0.001	—	—
	2016–2019	1.027 (−0.143, 2.212)	0.081	—	—
	2019–2021	−2.848 (−3.974, −1.709)	< 0.001	—	—
	1990–2021	—	—	2.254 (2.099, 2.410)	< 0.001

Age-standardized mortality rate	1990–1996	−0.289 (−0.519, −0.059)	0.016	—	—
	1996–2001	−1.035 (−1.461, −0.606)	< 0.001	—	—
	2001–2016	1.738 (1.673, 1.803)	< 0.001	—	—
	2016–2019	−0.889 (−2.233, 0.473)	0.186	—	—
	2019–2021	−4.039 (−5.340, −2.719)	< 0.001	—	—
	1990–2021	—	—	0.257 (0.088, 0.428)	< 0.001

**Table 2:** APC model analysis results of prostate cancer mortality among Iranian men.

Factor	Coef.	SE	95% CI	*p* value
Age (years)				
20–24	−3.409	0.176	−3.755, −3.064	< 0.001
25–29	−4.014	0.196	−4.400, −3.628	< 0.001
30–34	−3.170	0.129	−3.424, −2.916	< 0.001
35–39	−2.680	0.104	−2.885, −2.476	< 0.001
40–44	−2.322	0.090	−2.500, −2.144	< 0.001
45–49	−1.428	0.069	−1.564, −1.291	< 0.001
50–54	−0.503	0.055	−0.612, −0.395	< 0.001
55–59	0.116	0.045	0.027, 0.205	0.011
60–64	0.840	0.036	0.769, 0.912	< 0.001
65–69	1.349	0.029	1.292, 1.407	< 0.001
70–74	1.825	0.023	1.780, 1.871	< 0.001
75–79	2.306	0.020	2.266, 2.345	< 0.001
80–84	2.608	0.021	2.566, 2.650	< 0.001
85–89	2.805	0.026	2.753, 2.857	< 0.001
90–94	2.892	0.034	2.825, 2.960	< 0.001
+95	2.784	0.049	2.688, 2.880	< 0.001
Period				
1992–1996	−0.611	0.025	−0.661, −0.562	< 0.001
1997–2001	−0.423	0.017	−0.458, −0.389	< 0.001
2002–2006	−0.164	0.011	−0.185, −0.142	< 0.001
2007–2011	0.133	0.010	0.113, 0.153	< 0.001
2012–2016	0.428	0.015	0.398, 0.458	< 0.001
2017–2021	0.638	0.022	0.593, 0.683	< 0.001
Cohort				
< 1901	2.302	0.122	2.061, 2.543	< 0.001
1902–1906	2.069	0.072	1.927, 2.210	< 0.001
1907–1911	1.797	0.059	1.680, 1.914	< 0.001
1912–1916	1.469	0.044	1.381, 1.556	< 0.001
1917–1921	1.317	0.033	1.251, 1.383	< 0.001
1922–1926	1.120	0.028	1.064, 1.176	< 0.001
1927–1931	0.897	0.027	0.843, 0.952	< 0.001
1932–1936	0.687	0.030	0.627, 0.746	< 0.001
1937–1941	0.497	0.034	0.428, 0.565	< 0.001
1942–1946	0.265	0.041	0.183, 0.346	< 0.001
1947–1951	0.043	0.048	−0.051, 0.139	0.370
1952–1956	−0.199	0.056	−0.310, −0.088	< 0.001
1957–1961	−0.409	0.064	−0.536, −0.282	< 0.001
1962–1966	−0.681	0.074	−0.828, −0.534	< 0.001
1967–1971	−0.940	0.086	−1.110, −0.771	< 0.001
1972–1976	−1.154	0.101	−1.353, −0.955	< 0.001
1977–1981	−1.391	0.123	−1.633, −1.149	< 0.001
1982–1986	−1.593	0.142	−1.872, −1.314	< 0.001
1987–1991	−1.819	0.188	−2.189, −1.449	< 0.001
1992–1996	−2.029	0.286	−2.591, −1.467	< 0.001
1997–2001	−2.249	0.449	−3.130, −1.367	< 0.001
Akaike information criterion (AIC)	7.733			
Bayesian information criterion (BIC)	−247.525			

## Data Availability

The data that support the findings of this study are available from the corresponding author upon reasonable request.

## Nomenclature

PC: Prostate cancer
ASR: Age-standardized incidence rate
HDI: Human development index
GDP: Gross domestic product
PSA: Prostate-specific antigen screening
IARC: International Agency for Research on Cancer
APC: Age-period-cohort
GBD: Global Burden of Disease
ASMR: Age-standardized mortality rate
AAPC: Average annual percentage change
APCh: Annual percentage change
IE: Intrinsic estimator
RR: Relative risk
AIC: Akaike information criterion
BIC: Bayesian information criterion
URSPC: European Randomized Study of Screening for PC
